# The effect of the combination of whole body vibration and shoe with an unstable surface in chronic ankle instability treatment: a randomized clinical trial

**DOI:** 10.1186/s13102-021-00256-6

**Published:** 2021-03-19

**Authors:** Farideh Shamseddini Sofla, Mohammad Hadadi, Iman Rezaei, Negar Azhdari, Sobhan Sobhani

**Affiliations:** 1 Student Research Committee, School of Rehabilitation Sciences, Shiraz University of Medical Sciences, Shiraz, Iran; 2 Orthotics and Prosthetics Department, School of Rehabilitation Sciences, Shiraz University of Medical Sciences, Shiraz, Iran; 3 Rehabilitation Sciences Research Center, Shiraz University of Medical Sciences, Shiraz, Iran; 4 Physical Therapy Department, School of Rehabilitation Sciences, Shiraz University of Medical Sciences, 1 Abivardi Avenue, Chamran Blvd., Shiraz, 71345-1733 Iran

**Keywords:** Whole body vibration, Unstable shoe, Chronic ankle instability, Proprioception

## Abstract

**Background:**

Chronic ankle instability (CAI) is a common condition following an ankle sprain. This study investigated the effects of whole body vibration (WBV) and shoe with an unstable surface training on balance, functional performance, strength, joint position sense in people with CAI.

**Method:**

Thirty- four peoples with unilateral CAI were randomly assigned to three groups: WBV group, WBV with shoe with an unstable surface (WBV-S), and no treatment control group (CON). The WBV group received 4 weeks progressive WBV training and the WBV-S group received progressive WBV training with shoe with an unstable surface. Modified star excursion balance test (mSEBT)reach distance, Hop-Test, muscle strength, and joint position sense were measured at baseline and after the 4 weeks; Moreover, the mSEBT and Hop-Test were reassessed again 2 weeks post intervention.

**Results:**

The result showed a significant group-by-time interaction for anterior and posterolateral directions of mSEBT. The reach distance of these directions at post-intervention and follow-up increased significantly compare to pre-intervention in the WBV and WBV-S groups but not significantly change in the CON group. The Hop test in the WBV-S group was significantly more at post-intervention and follow-up compared to pre-intervention. However, no significant changes were observed in WBV and CON groups. No significant changes were observed for mSEBT posteromedial direction, muscles strength, and joint position sense errors.

**Conclusion:**

The 4 weeks WBV and WBV-S interventions could improve balance in peoples with CAI. Improvement in Hop test was only observed in the WBV-S group suggesting the added value of combining WBV and shoe with an unstable surface as an effective therapy compared to WBV training alone. The use of WBV and WBV-S were not associated with significant changes in strength and joint position sense variables over a four-week period.

**Trial registration:**

This work registered in the Iranian Registry of Clinical Trials (IRCT20151118025105N4).

## Introduction

Ankle sprains remain a significant injury in both athletes and non-athletes groups, and 32 to 74% of injured people will eventually develop chronic ankle instability (CAI) [1]. According to the International Ankle Consortium, the CAI “has been defined in a variety of ways, but is most predominantly described as an encompassing term used to classify a subject with both mechanical and functional instability of the ankle joint” [2]. The people with CAI usually experience a range of neuromuscular deficits such as reduced muscle strength [3], impaired proprioception [3], and balance [4] control leading to significant functional limitations [5]. These factors also make people with the CAI more susceptible to reinjury which significantly impacts people’s quality of life in the long-term [3, 6, 7].

Balance training such training on unstable surface is used and emphasized as one of the most effective interventions in various stages of CAI rehabilitation [8, 9]. This training increases muscle co-contraction, stiffness, joint stability, balance [10], and improving mechanoreceptors. Various devices are used for balance training exercises such as balance boards [11] foam rollers, trampolines [8], unstable surface [12, 13], and whole body vibration (WBV).

The use of WBV has gained popularity for a number of years [14]. The WBV is a neuromuscular training that transmission of mechanical oscillations from a vibrating platform lead to activating the primary endings of the muscle spindles, joint mechanoreceptor, the excitability of alpha and gamma motor neurons, brain activity, and strength [13, 15, 16]. These physiological changes could lead to more effective proprioceptive feedback, thereby improving balance ability and the active protection mechanism of the ankle joint [17].The peoples with CAI have neuromuscular and proprioceptive deficits [18]. Because of the strong sensory stimulus and activation of the alpha-motor neurons, WBV training may also enhance strength [19] proprioception [20], and balance in ankle instability [21].

Unstable shoes have the potential to be used as a unstable surface to improve neuromuscular system problems [22]. Unstable shoes have a soft and rounded sole that provides an unstable base in the anterior-posterior and medial-lateral directions [23]. Some studies have indicated that unstable shoes improve balance in long-term use [23], increase the electromyography of the ankle muscles [24], increase the sensory feedback [25], and reduce the joints pain [26]. Unstable shoes can affect the biomechanics of the lower extremity, so they can challenge the stability of the body and involve the ankle and hip muscles to maintain mediolateral stability [27].

Considering the positive effects of WBV, unstable shoes, and unstable surfaces on neuromuscular factors, some researchers have already tried to combine both interventions and investigate the combined effects of these two modalities [13, 15, 28].

According to previous findings, it is of interest to evaluate the combined use of WBV and unstable surfaces as a means to improve balance and its components [15, 28]. Previously, Sierra et al. showed that training on unstable surface on the WBV improved the reaction time of the peroneus brevis, peroneus longus, and Tibialis anterior muscles in the peoples with CAI [28]. Sierra et al. in another study show that these combinations can improve static and dynamic balance in the peoples with CAI [13]. Furthermore, Cloak et al. investigated that the combination of these methods can improve center of mass distribution and the results of some functional balance tests, in athletes with CAI [15]. These studies showed that the WBV exercise and training on unstable surface have a positive effect on balance so when they are combined with each other, effectiveness of training may increase. Working with these combinations, however, seems to be challenging because the training surface can become too unstable for use in the peoples with CAI.

In mentioned studies [13, 15, 28] did not use unstable shoes and also did not compare effect of such combined treatment with vibration only. The use of shoes with an unstable surface may be an alternative to wobble/balance boards because of their main advantage of being attached to the body, which leads to a lower instability condition. In addition, the use of such shoes may help to localize the effect of WBV on the ankle joint and improve the outcome of treatment.

The purpose of the present research was, therefore combination of WBV and shoe with an unstable surface (WBV-S) on dynamic balance, functional performance, muscle strength, and joint position sense in the peoples with CAI. We hypothesized that the combination of WBV and shoe with an unstable surface is more effective than WBV alone to improve the mentioned clinical outcomes in the people with CAI.

## Material and methods

### Study design

This randomized control trial was conducted from April 2019 to September 2019. The protocol of this study was approved by the local ethics committee (IR.SUMS.REHAB.REC.1397.005) in accordance with the Declaration of Helsinki and registered in the Iranian Registry of Clinical Trials (IRCT20151118025105N4). All individuals gave written informed consent before participation. The participants were randomly assigned to one of the following three groups (1:1:1 ratio): WBV-S, WBV, and control (CON). The participants were randomized via the permuted block randomization method with a block size of nine. Data collection procedures were done at the Faculty of Rehabilitation Sciences of Shiraz University of Medical Sciences.

### Participants and setting

Forty-five volunteer peoples with unilateral CAI participated in this study. The inclusion and exclusion criteria for peoples with CAI were based on the International Ankle Consortium Position Statement [2]. The inclusion criteria were; a history of at least one significant ankle sprain with the first sprain occurring at least 12 months before the study, the sprain associate with inflammatory symptoms (e.g. pain, swelling, etc.) and interrupt desired physical activities for at least 1 day, participant’s report of their ankle joint giving way and/or recurrent ankle sprains (at least two episodes 6 months before the study), feeling of instability confirmed by a Cumberland Ankle Instability Tool (CAIT) score < 24, and a score of < 90% in the daily activities section and < 80% in the sports activities section of the Foot and Ankle Ability Measure (FAAM) questionnaire. The participants provided information with the Persian versions of the CAIT and FAAM questionnaire [29, 30]. The exclusion criteria were as follows; a history of previous surgery in the musculoskeletal system in either or both lower limbs, history of fracture needing realignment in either or both lower limbs, acute injury to other lower limb joints which interrupted the desired physical activities for at least 1 day, potential contraindication of WBV usage (e.g. kidney stone, acute disc herniation, etc.), and other diseases due to balance problem (e.g. peripheral neuropathy, MS, Parkinson, Migraine, Radiculopathy, middle ear disease).

### Interventions

#### WBV group

All participants in this group received the vertical type WBV machine (Fitvibexcel pro, uniphy, ElektromedizinGmbH & Co. KG– Germany) training for 12 sessions. All training sessions where held from 12.00 a.m. to 4.00 p.m. once a day, 3 times a week, for 4 weeks. The progressive program consisted of increasing duration and frequency of vibration [31]. The amplitude was 3 mm and the frequency of the WBV increased from 30 Hz to 40 Hz and duration increased from 35 s to 60 s. Each session contained three sets and the rest between each bout of training was 45 s. The participants performed the protocol training while standing on both feet. To localize the vibration on the ankle and reduce the vibration transmission to the trunk, the participants were asked to stand on the platform in a semi-squat position (30° of knee flexion) and weight was posed on the forefoot [31, 32]. To standardized knee flexion degree between the sessions the physical therapist who supervised the intervention sessions asked the participants to bend their knees as if they were going to sit down and their knees did not go over their toes. It was possible for the participants to stop the WBV machine by pressing the emergency stop switch. Moreover, to prevent probable falling, the physical therapist stood at the back of the WBV machine during the training session.

#### WBV-S group

**Fig. 1:**
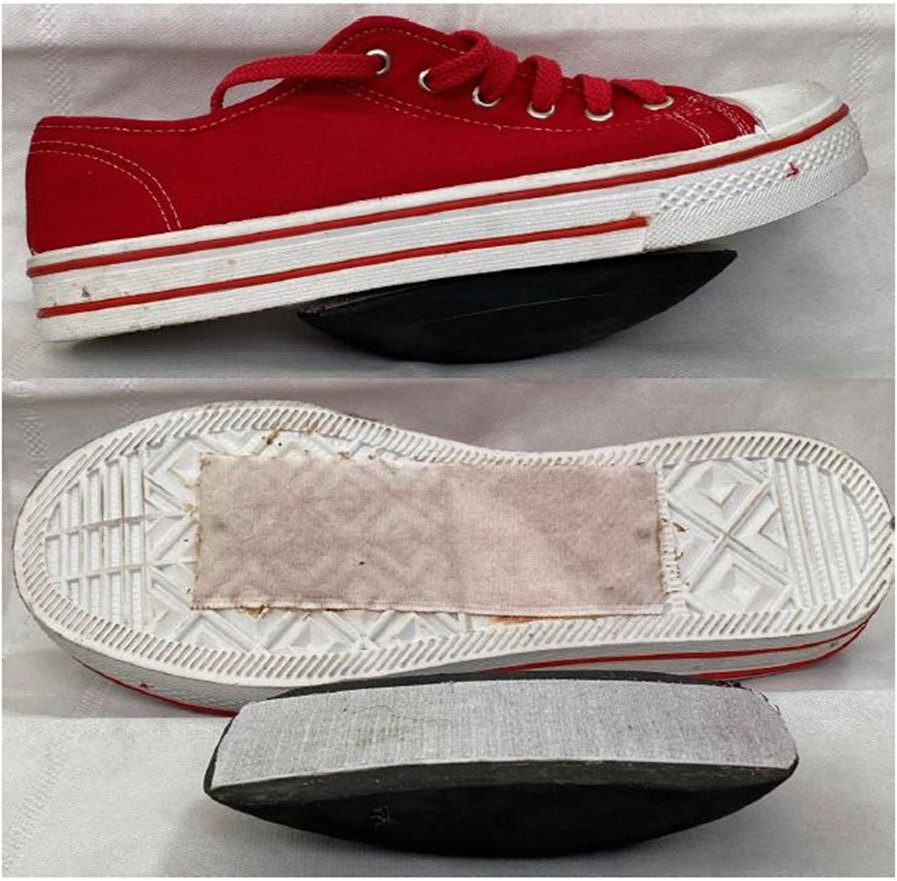
Shoe with an unstable surface

The participants in this group received WBV with the same parameter and duration as the first group while they wore the shoe with an unstable surface. The detachable rocker of the shoe was made of ethylene-vinyl acetate of 15 mm thickness with standard hardness (shore-A 30–40). It was designed for each participant based on his/her shoe size. This rocker has anterior-posterior and medial-lateral curvatures and covers 60% of shoe length. The apex of the rocker was at the midpoint of the shoe so that it formed a semispherical with 100-degrees curvature (Fig. 1).

#### CON group

The CON group received no intervention between the initial and final assessments. All participants in this group received 10 sessions of routine physiotherapy as appropriate for their conditions after following up assessment.

All interventions were carried out by an expert physical therapist.

### Outcome measures

Our primary outcome was the dynamic balance assessed using the modified Star Excursion Balance Test (mSEBT) [33]. The secondary outcomes included functional performance, muscle strength, and joint position sense. To assess the functional performance we used the single-leg hop for distance test (Hop-Test) [34]. The muscle strength and joint position sense were assessed by using an isokinetic dynamometer®(Biodex System 4 pro, Biodex Medical System Inc., Shirley, New York) [35]. All outcomes were measured at baseline and after 4 weeks (post-intervention). Moreover, the mSEBT and Hop-Test were reassessed again 2 weeks post intervention. All participants performed 10-min cycling for warming up and cooling down, consisting of mild pedalling on an ergometric bicycle and ballistic [36]. All assessment procedures were done under the same conditions. The order of the test was as follows: 1) mSEBT, 2) Hop-Test, 3) muscle strength, and 4) joint position sense. The rest period was considered 5 minutes between various tests.

#### mSEBT

The mSEBT is a modified and simplified version of the SEBT that uses three directions (anterior, posteromedial, and posterolateral). The test is reliable [37] and the most effective measurement instrument to assess dynamic balance in the peoples with CAI [33]. The angle between the anterior axis and the other two posteromedial and posterolateral axes is 135 degrees. The posteromedial and posterolateral axes make an angle of 90 degrees with each other. All participants had to stand on their affected leg with their great toe located at the center of the point where the three axes of the Y met. They were asked to use their unaffected leg to reach as far as possible in all three directions. For each direction, the participants performed six practice trials and three test trials [33]. Two minutes rest time was considered between each trial. Trials were discarded and repeated if the stance foot moved or lifted from the floor, the reach foot failed to return to the starting position or was used for support, and hands were removed from the hips. The distance reached was recorded in centimeters and the average of the three measurements normalized to lower extremity length for further analysis. The leg length was measured from the anterior superior iliac spine to the inferior margin of the medial malleolus.

#### Hop-test

This Hop-Test is a valid measure of the lower extremity functional performance [34]. The participants stood on the affected leg with the great toe on the starting line and their unaffected leg flexed but not touching the weight-bearing leg. They were asked to perform three maximal hops forward while arms swing was allowed. The test was considered successful if the participant was able to land in a controlled, stable manner. The participants performed three practice trials to get familiar with the test and then performed three test trials. Rest time was considered 2 minutes between each trial. The test trial was repeated if the participant could not hop without losing balance and contacting the ground with the unaffected leg. The distance from the start to the correct point where the heel struck the ground was recorded in centimeters. The maximum distance was used for further analysis.

#### Muscle strength and joint position tests

The muscle strength and joint position sense were both assessed using an isokinetic dynamometer. This measurement tool is reliable and valid [35]. The concentric and eccentric strength of ankles eversion-inversion movements were tested at 40 degrees range of motion (15 degrees eversion to 25 degrees inversion). The participants sat on the isokinetic dynamometer chair, while the involved ankle was placed on the inversion-eversion attachment and the backrest at an angle of 70 degrees. The lower leg was parallel to the floor with an 80–110 degrees flexion position of the knee while the trunk, leg, and foot were fixed by a strap. The rotation axis of the dynamometer was level at the subtalar joint. The subtalar joint was in a natural position and it was ensured through palpation of the dome of the talus. The participants performed three submaximal practical tests. Sixty seconds was considered for rest and then they performed three maximum trial tests at a speed of 90 degrees per second. The participants were encouraged verbally for maximum effort [36]. The peak torque per body-weight of the invertor and evertor ankle muscles strength during concentric and eccentric contraction was recorded for further analysis.

To assess joint position sense, the participant position was similar to muscle strength testing. Active and passive joint position sense was assessed at five and 15 degrees inversion. For the passive test, the participants’ foot was moved to maximum eversion passively and then to five degrees or 15-degree inversion randomly and held for 10s. The foot was brought to maximum eversion and moved back passively to inversion at a speed of four degrees per second. The participant pushed a stop bottom when she or he reached the test position. The active test was performed similarly, except that the participant was asked to move back the foot to the test position actively and push the stop bottom when he/she reached the position test. The participants performed three practical tests and three test trials for the two test positions. The participants were blindfolded throughout the examination [3]. In this study, the mean of absolute error was recorded for further analysis. The absolute error is the difference in absolute value in degrees between the position chosen by the subject and the test-position angle. This protocol was similar to previous studies [3, 38].

### Blinding

During the study a physical therapist supervised the intervention sessions and another one who was blinded to group assignments assessed the outcomes. The allocation procedure was concealed from the outcomes assessor by using sequentially numbered, opaque, sealed, and stapled envelopes. The person responsible for randomization was different from the outcome assessor and the therapist. Statistical analyses were carried out by a statistician blinded to the participant allocation. The randomization codes were decoded only after the completion of the data analysis.

### Statistical method

Based on the mSEBT anterior reach distance data of a similar study [15], and assuming 85% power, α = .05 and effect size of 1.05, we estimated the sample size as 33 (11 in each group). In anticipation of an overall attrition rate of 25%, we increased the final sample size to 45 (15 in each group).

All data were analyzed using SPSS software (version 21; SPSS, Inc., Chicago, IL, USA). The normality of variables was tested using Kolmogorov–Smirnov test initially and all variables were presented as a normal distribution. In this study, we used the modified intention to treat with simple imputation, included all randomized participant who had at least one baseline measurement [39]. Each outcome measure was analyzed using repeated-measures ANOVA with 1 between-subject factor (intervention) and 1 within-subject factor (time). Identification of a significant interaction between time and group led to further analysis. In this regard to assess the time effectiveness within each group separate repeated measure ANOVA tests were performed. The post hoc tests used LSD correction to adjust for multiple comparisons. The level of significance was set at *P*-value less than .05.

## Results

**Fig. 2:**
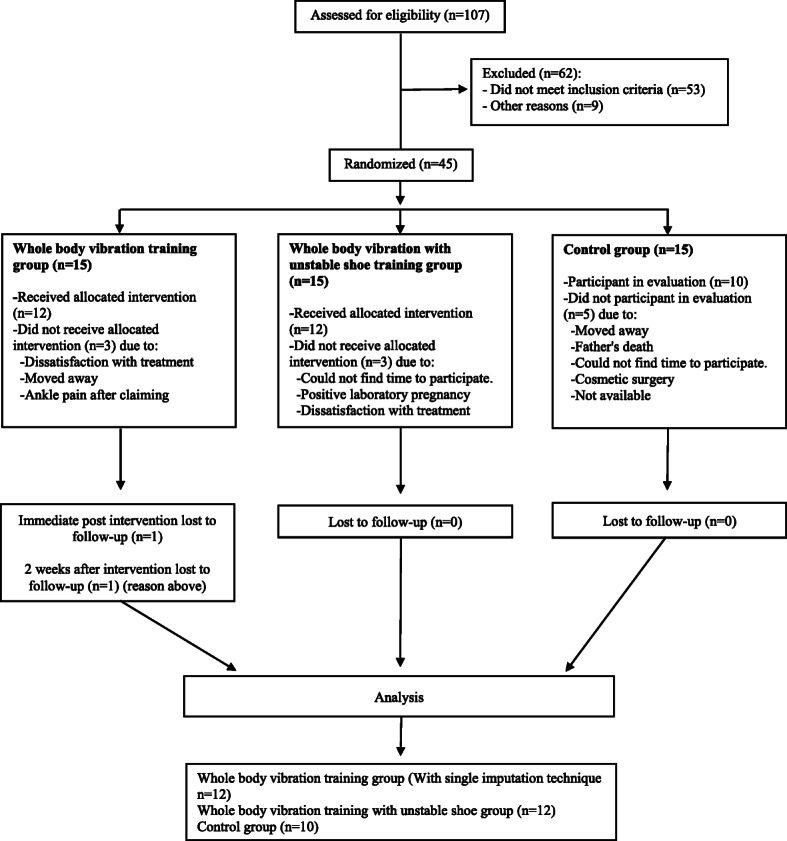
Flow diagram showing participant flow and follow-up evaluation

Among the 45 participants enrolled in the trial, 34 participants completed the study. The CONSORT Statement flow diagram is shown in Fig. 2. The demographic and clinical data of the participants are summarized in Table 1. There were no differences among the study groups in demographic and clinical data.

**Table 1:** Baseline demographic and clinical characteristics in each group

Variable	WBVS (*n* = 12)	WBV (*n* = 12)	CON (*n* = 10)
Gender (female n)	8	8	5
Age (years)	40.58 (8.76)	35.83 (12.08)	38.40 (10.49)
Height (cm)	163.91 (8.53)	164.29 (12.54)	166.20 (9.50)
Weight (kg)	74.88 (11.48)	71.61 (13.93)	73.60 (9.10)
Affected limb (right n)	8	7	7
Cumberland ankle instability tool (score)	10.41 (5.45)	10.58 (3.44)	9.40 (4.45)
Foot and ankle ability measure (score %)			
Sport	62.41 (19.85)	70.30 (17.65)	70.78 (20.32)
Activity daily living	58.93 (15.20)	60.93 (17.65)	52.60 (20.32)
Modified star excursion balance test (%)			
Anterior	59.95 (7.86)	62.99 (10.02)	60.04 (8.07)
Posterolateral	69.49 (19.12)	69.54 (17.23)	73.89 (14.87)
Posteromedial	90.77 (11.68)	87.14 (13.90)	90.10 (17.53)
Hop-test (cm)	47.49 (19.76)	48.62 (20.40)	53.49 (33.52)
Peak torque (nm/kg)			
Concentric evertor	23.41 (7.12)	20.15 (5.32)	24.75 (17.29)
Eccentric evertor	33.55 (9.08)	28.88 (9.33)	33.45 (13.21)
Concentric invertor	32.07 (7.32)	24.66 (10.33)	31.96 (16.34)
Eccentric invertor	31.96 (17.89)	28.50 (9.83)	31.45 (16.35)
Joint position sense error (degrees)			
Active 5°	2.85 (1.46)	3.53 (1.53)	3.34 (1.61)
Active 15°	4.54 (2.13)	4.36 (1.39)	2.26 (1.13)
Passive 5°	3.68 (1.27)	3.65 (1.30)	3.80 (1.47)
Passive 15°	4.82 (1.58)	4.34 (2.38)	3.31 (2.28)

Note: Values are means and (Standard Deviation)

*Abbreviations*: *WBV* Whole body vibration group, *WBVS* Whole body vibration with shoe with an unstable surface group, *CON* Control group, *n* number

**Fig. 3:**
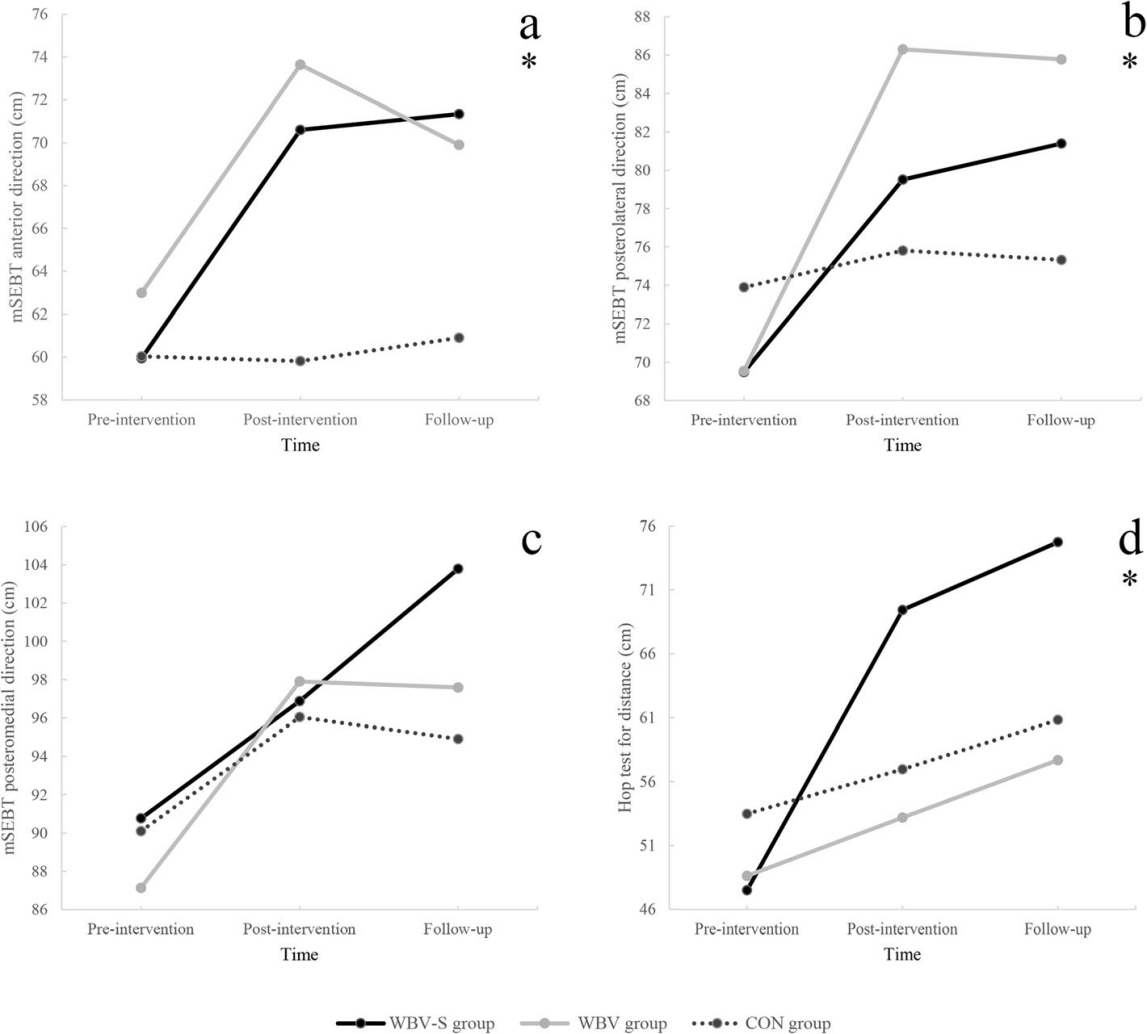
Interactions between group and time for mSEBT (%) in (**a**) anterior (**b**) posterolateral and (**c**) posteromedial directions and (**d**) hop test for distance (cm). Abbreviations: mSEBT, Modified star excursion balance test; WBV, Whole body vibration group; WBV-S, Whole body vibration with shoe with an unstable surface group; CON, Control group. Note: Values are means and (Standard Deviation). *Significant interaction between group and time

A significant group-by-time interaction was observed for anterior (*F*_2, 22_ = 8.45, *P* = .002) and posterolateral (*F*_2, 22_ = 4.56, *P* = .02) directions of mSEBT (Fig. 3). Comparison of times effects in each group showed a significant increase in reach distance of these directions at post-intervention and follow-up compare to pre-intervention in the WBV and WBV-S groups but not significantly change in the CON group (Table 2). Group-by-time interaction (*F*_4, 62_ = 1.92, *P* = .10) and between-group comparison were not significant for mSEBT posteromedial direction.

**Table 2:** Within-group and between-group effects for variables (posteromedial direction of modified star excursion balance, peak torque, and Joint position sense error) without significant interaction

Variable	Group	Pre-intervention	Post-intervention	Follow-up	Within-group		Between-group	
					F	P	F _**2, 31**_	P
**Modified star excursion balance test (%)**								
Posteromedial	WBV-S	90.77 (11.68)	96.88 (13.96)	103.79 (15.39)	F _2, 62_ = 19.42	< 0.001 †	0.20	0.81
	WBV	87.14 (13.90)	97.90 (18.21)	97.58 (14.64)				
	CON	90.10 (17.53)	96.04 (14.74)	94.91 (13.38)				
**Peak torque (nm/kg)**								
Concentric evertor	WBV-S	23.41 (7.12)	26.26 (8.01)	NA	F _1, 31_ = 0.47	0.49	2.61	0.08
	WBV	20.15 (5.31)	15.70 (6.12)	NA				
	CON	24.75 (17.29)	22.65 (9.15)	NA				
Eccentric evertor	WBV-S	33.55 (9.08)	32.96 (8.79)	NA	F _1, 31_ = 0.03	0.85	0.56	0.57
	WBV	28.88 (9.33)	30.49 (8.85)	NA				
	CON	33.45 (13.21)	33.16 (11.92)	NA				
Concentric invertor	WBV-S	32.07 (7.32)	31.75 (9.96)	NA	F _1, 31_ = 0.02	0.87	1.04	0.36
	WBV	24.66 (10.33)	26.70 (10.44)	NA				
	CON	31.69 (16.34)	29.70 (14.65)	NA				
Eccentric invertor	WBV-S	31.96 (7.89)	32.13 (9.65)	NA	F _1, 31_ = 0.38	0.53	2.00	0.15
	WBV	28.50 (9.83)	24.03 (3.73)	NA				
	CON	31.45 (16.35)	32.03 (18.03)	NA				
**Joint position sense error (degrees)**								
Active 5°	WBV-S	2.85 (1.46)	4.11 (2.15)	NA	F _1, 31_ = 3.47	0.06	0.96	0.39
	WBV	3.53 (1.53)	4.61 (2.40)	NA				
	CON	3.34 (1.61)	3.22 (1.19)	NA				
Active 15°	WBV-S	4.54 (2.13)	3.45 (2.50)	NA	F _1, 31_ = 1.08	0.30	2.34	0.11
	WBV	4.36 (1.39)	3.92 (1.81)	NA				
	CON	2.26 (1.13)	3.07 (1.31)	NA				
Passive 5°	WBV-S	3.68 (1.27)	2.61 (2.08)	NA	F _1, 31_ = 9.71	< 0.001 †	0.13	0.87
	WBV	3.65 (1.30)	2.62 (0.92)	NA				
	CON	3.80 (1.47)	2.91 (1.07)	NA				
Passive 15°	WBV-S	4.82 (1.58)	2.99 (2.49)	NA	F _1, 31_ = 4.29	0.05	0.17	0.84
	WBV	4.34 (2.38)	3.33 (1.41)	NA				
	CON	3.31 (2.28)	3.70 (1.78)	NA				

Note: Values are means and (Standard Deviation). Significant post hoc difference between: † pre- and post-intervention

*Abbreviations*: *WBV* Whole body vibration group, *WBV-S* Whole body vibration with shoe with an unstable surface group, *CON* Control group, *NA* Not applicable

The result of repeated measure ANOVA showed a significant group-by-time interaction (*F*_1.25, 13.84_ = 10.38, *P* = .004) for Hop-Test (Fig. 3). Based on the results of further analysis, the participants in the WBV-S group hop significantly more at post-intervention and follow-up compared to pre-intervention. However, no significant changes were observed in WBV and CON groups at post-intervention and follow-up (Table 2).

**Fig. 4:**
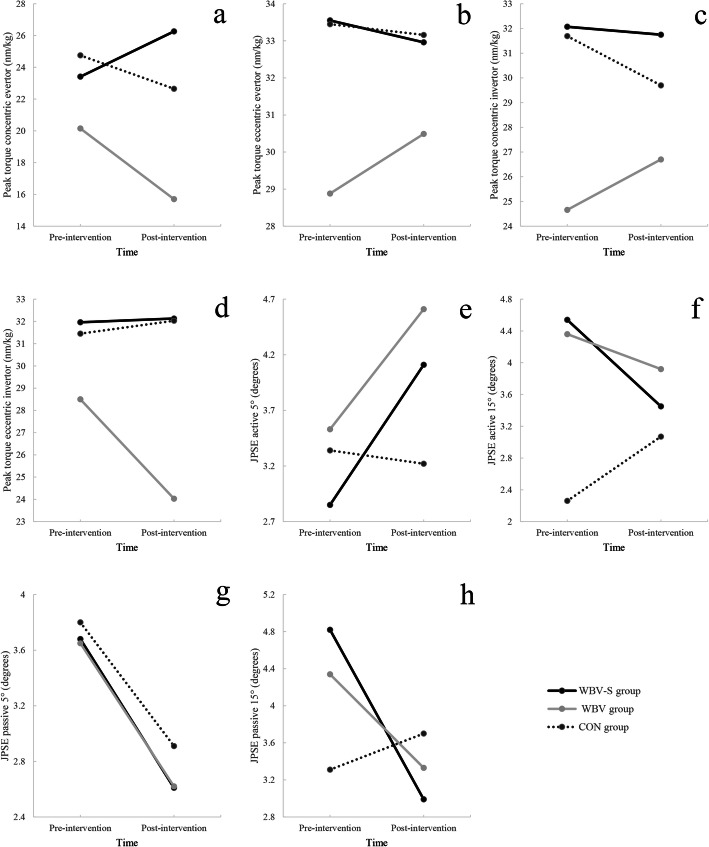
Interactions between group and time for peak torque (nm/kg) (**a**) concentric evertor (**b**) eccentric evertor (**c**) concentric invertor and (**d**) eccentric invertor, active JPSE (degrees) in (**e**) five-degree inversion and (**f**) 15-degree inversion, passive JPSE (degrees) in (**g**) five-degrees inversion and (**h**) 15-degree inversion. Abbreviations: JPSE, joint position sense error; WBV, Whole body vibration group; WBV-S, Whole body vibration with shoe with an unstable surface group; CON, Control group. Note: Values are means and (Standard Deviation)

No significant group-by-time interactions were observed for muscles strength [i.e. concentric evertors (*F*_2, 31_ = 1.53, *P* = .23), eccentric evertors (*F*_2, 31_ = 0.61, *P* = .76), concentric invertors (*F*_2, 31_ = 1.27, *P* = .29), eccentric invertors (*F*_2, 31_ = 0.67, *P* = .51)] (Fig. 4). Moreover, within- and between-group comparisons were not significant in any of these variables (Table 3).

**Table 3:** Separate repeated measure ANOVA tests for variables (anterior and posterolateral directions of modified star excursion balance test, and hop-test) with significant interaction to compare time effect in each group

Variable	Group	Pre-intervention	Post-intervention	Follow-up	F	***P***
**Modified star excursion balance test (%)**						
Anterior	WBV-S	59.95 (7.86)	70.61 (11.73)	71.35 (13.12)	F _2, 22_ = 8.45	0.002 † ‡
	WBV	62.99 (10.02)	73.64 (11.42)	69.91 (10.53)	F _2, 22_ = 8.32	0.002 † ‡
	CON	60.04 (8.07)	59.82 (5.30)	60.91 (3.59)	F _1.22, 11.02_ = 0.20	0.71
Posterolateral	WBV-S	69.49 (19.12)	79.51 (23.89)	81.40 (23.26)	F _2, 22_ = 4.56	0.02 † ‡
	WBV	69.54 (17.23)	86.29 (18.37)	85.77 (16.43)	F _1.42, 15.63_ = 15.45	< 0.001 † ‡
	CON	73.89 (14.87)	75.81 (20.69)	75.32 (17.70)	F _2., 18_ = 0.10	0.9
**Hop-test (cm)**						
	WBV-S	47.49 (19.76)	69.44 (37.62)	74.75 (40.30)	F _1.25, 13.84_ = 10.38	0.004 † ‡
	WBV	48.62 (20.40)	53.19 (19.85)	57.70 (23.39)	F _2, 22_ = 1.52	0.24
	CON	53.49 (33.52)	56.98 (33.52)	60.82 (33.54)	F _2, 18_ = 1.79	0.19

Note: Values are means and (Standard Deviation). Significant post hoc difference between: † pre- and post-intervention; ‡ pre-intervention and follow-up

*Abbreviations*: *WBV* Whole body vibration group, *WBV-S* Whole body vibration with shoe with an unstable surface group, *CON* Control group

Also, the results revealed no significant group-by-time interactions for passive and active joint position sense errors in evaluated angles [i.e. active joint position sense in five degrees inversion (*F*_2, 31_ = 1.20, *P* = .310), active joint position errors in 15 degrees inversion (*F*_2, 31_ = 1.61, *P* = .21), passive joint position errors in five degrees inversion (*F*_2, 31_ = 0.02, *P* = .97), passive joint position errors in 15 degrees inversion (*F*_2, 31_ = 2.59, *P* = .09). In addition, except of within group comparison of passive joint position errors in five degrees inversion (*F*_1, 31_ = 9.71, *P* < 0.001), there was no significant difference in between- and within-group comparisons of joint position error variables (Table 3).

## Discussion

The mSEBT reach distance was considered as the primary outcome in the current study. The results indicated that the anterior and posterolateral reach distance of mSEBT improved in both WBV and WBV-S groups post-intervention. There was no significant improvement in the posteromedial reach of mSEBT in all groups. At least 6–8% changes in normalized scores of SEBT are needed to feel confident that a real change has occurred [40]. In both intervention groups, WBV and WBV-S, the improvement of reach distances in all directions were greater than these values. This may indicate that both interventions could be equally effective in the improvement of dynamic balance in the people with CAI. These results are in line with previous studies. Sierra et al. indicated that 6-week progressive WBV training combined with BOSU® Balance Trainer as unstable surface improved medial, posterolateral, and composite reach of SEBT after the intervention [13]. Also, Cloak et al. suggested that a 6 weeks combination of the an unstable surface with vibration training (vibrosphere) improved mSEBT, in football player with chronic ankle instability [15].

It is, however, interesting to find that the treatment gains in dynamic balance were maintained at a 2 weeks follow up. This is in contrast to the finding of Sierra et al. that showed the values tended to return to baseline levels in the medial and posteromedial directions and the composite score of SEBT after a 6-week follow up, in recreational athletes with CAI [13]. This may be due to differences in the type of unstable surface of the current study and its shorter follow-up duration in comparison to the Sierra study. Although they investigate different population, the present result is in line with the findings of Sobhani et al. who studied the combined effect of WBV-S on balance measures in older adults. They found maintenance in balance improvement even at one-month post-intervention in the combined group [41].

Our hypothesis of clinical superiority of WBV-S vs WBV alone were not supported for dynamic balance measure, one of the reasons may be insufficient duration of treatment.

Regarding function, the results showed that the WBV-S improved Hop-Test distance and this improvement was maintained at 2 weeks follow-up while no improvement was found by WBV training alone. This finding was in line with our hypothesis. In similar Cloak et al. reported improvements in the Hop-Test measure in people with CAI who trained with an unstable vibrating surface [15]. Performing the Hop-Test requires an adequate level of strength, neuromuscular coordination, and joint stability. WBV training could enhance muscle spindle sensitivity and excitability of the alpha and gamma motoneurons. These neuromuscular adaptations lead to the reduced reaction time of the ankle joint stabilizer muscles and the motor-unit recruitment thresholds [17].

A previous study showed that wearing an unstable footwear was associated with greater ankle muscle activity [42]. The greater muscular activity compensates for the instability induced by unstable surface. This could increase demands on the sensorimotor system and may induce effective training stimuli to improve postural control [12].

Also, these information might indicate that adding an unstable surface to WBV training, due to using the proprioceptive feedback loop generated by enhanced intramuscular force and isometric control, could provide more effects [43].

In this study, we did not observe any significant changes in concentric-eccentric eversion and inversion peak torque in any group. These results were in line with those obtained by Sierra et al. [28]. They examined the effect of combined WBV and unstable surface on the peak torque, electrical activity, and reaction time of the ankle muscle in the people with CAI and found no improvement in evertor ankle muscles strength. The lack of improvement in isokinetic strength might be associated with the issue of mode specificity [44]. Our interventions were delivered in a closed kinematic chain format, while the Biodex isokinetic testing procedures were assessed in an open kinematic chain. It is also likely that our interventions were not rigorous enough to cause significant changes in the frontal plane and thus improve eversion and inversion peak torques.

Our null hypothesis that joint position sense would be improved with the WBV training program and the WBV-S would be more effective than WBV training, was rejected too. The joint position sense was not significantly different between the groups and no improvement was found post-intervention and after the follow-up period. Similarly, Otzel et al. assessed joint position sense after WBV training in individuals with CAI. The result shows no improvement in the joint position sense. In contrast, Bogaerts et al. assessed joint position sense by sensory organization test and reported that 12 months WBV improved joint position sense in healthy people. It is difficult to compare their results with those of the current study because they used different assessment tools and interventions. The possible reason for the lack of significant joint position sense improvement is the negative WBV training effect on mechanoreceptors because of fatigue [45].

## Limitations and future work

This study was faced with some limitations. In the present study we designed detachable rocker to making same condition for two treatment groups, how to connect this rocker to the shoe may affect the efficacy of unstable surface.

## Conclusion

The results of this study suggested that a 4 weeks WBV and WBV-S interventions could improve dynamic balance in the people with CAI. Improvement in functional performance was only observed in the WBV-S group suggesting the added value of combining WBV and shoe with an unstable surface as an effective therapy compared to WBV training alone. The use of WBV and WBV-S were not associated with significant changes in muscles strength and joint position sense variables over a 4 weeks period.

## Data Availability

The datasets used and/or analysed during the current study are available from the corresponding author on reasonable request.

## Abbreviations

CAI: Chronic ankle instability
WBV: Whole body vibration
WBVS: Whole body vibration with shoe with an unstable surface
CON: Control
CAIT: Cumberland Ankle Instability Tool
FAAM: Foot and Ankle Ability Measure
mSEBT: Modified star excursion balance test
Hop-Test: Single leg hop for distance test
ROM: Range of motion

## References

[1] Gribble PA, Bleakley CM, Caulfield BM, Docherty CL, Fourchet F, Fong DT-P, Hertel J, Hiller CE, Kaminski TW, McKeon PO, Refshauge KM, Verhagen EA, Vicenzino BT, Wikstrom EA, Delahunt E (2016). Evidence review for the 2016 international ankle consortium consensus statement on the prevalence, impact and long-term consequences of lateral ankle sprains. Br J Sports Med.

[2] DE Gribble PA, Bleakley CM, Caulfield B, Docherty CL, Fong DT, Fourchet F, Hertel J, Hiller CE, Kaminski TW, McKeon PO (2014). Selection criteria for patients with chronic ankle instability in controlled research: a position statement of the International Ankle Consortium. J Athl Train.

[3] Willems T, Witvrouw E, Verstuyft J, Vaes P, De Clercq D (2002). Proprioception and muscle strength in subjects with a history of ankle sprains and chronic instability. J Athl Train.

[4] Hintermann B (1999). Biomechanics of the unstable ankle joint and clinical implications. Med Sci Sports Exerc.

[5] Buchanan AS, Docherty CL, Schrader J (2008). Functional performance testing in participants with functional ankle instability and in a healthy control group. J Athl Train.

[6] Jain TK (2014). Objective evaluation of functional ankle instability and balance exercise treatment.

[7] Lotito G, Pruvost J, Collado H, Coudreuse J-M, Bensoussan L, Curvale G, Viton JM, Delarque A (2011). Peroneus quartus and functional ankle instability. Ann Phys Rehabil Med.

[8] Cruz-Diaz D, Lomas-Vega R, Osuna-Pérez M, Contreras F, Martínez-Amat A (2015). Effects of 6 weeks of balance training on chronic ankle instability in athletes: a randomized controlled trial. Int J Sports Med.

[9] McKeon PO, Hertel J (2008). Systematic review of postural control and lateral ankle instability, part II: is balance training clinically effective?. J Athl Train.

[10] Hrysomallis C (2011). Balance ability and athletic performance. Sports Med.

[11] Gil-Gómez J-A, Lloréns R, Alcañiz M, Colomer C (2011). Effectiveness of a Wii balance board-based system (eBaViR) for balance rehabilitation: a pilot randomized clinical trial in patients with acquired brain injury. J Neuroeng Rehabil.

[12] Turbanski S, Lohrer H, Nauck T, Schmidtbleicher D (2011). Training effects of two different unstable shoe constructions on postural control in static and dynamic testing situations. Phys Ther Sport.

[13] Sierra-Guzmán R, Jiménez-Diaz F, Ramírez C, Esteban P, Abián-Vicén J (2018). Whole-body–vibration training and balance in recreational athletes with chronic ankle instability. J Athl Train.

[14] Holmes A, Delahunt E (2009). Treatment of common deficits associated with chronic ankle instability. Sports Med.

[15] Cloak R, Nevill A, Day S, Wyon M (2013). Six-week combined vibration and wobble board training on balance and stability in footballers with functional ankle instability. Clin J Sport Med.

[16] Moezy A, Olyaei G, Hadian M, Razi M, Faghihzadeh S (2008). A comparative study of whole body vibration training and conventional training on knee proprioception and postural stability after anterior cruciate ligament reconstruction. Br J Sports Med.

[17] Pollock RD, Woledge RC, Martin FC, Newham DJ (2012). Effects of whole body vibration on motor unit recruitment and threshold. J Appl Physiol.

[18] Hertel J (2000). Functional instability following lateral ankle sprain. Sports Med.

[19] Aminian Far A, Hedayati R, Bagheri P, Yaghubi Z. Effects of whole body vibration on concentric torque of ankle invertor and evertor muscles in people with functional ankle instability. Koomesh. 2016;18(2):286-94.

[20] Martínez F, Rubio JA, Ramos DJ, Esteban P, Mendizábal S, Jiménez F (2013). Effects of 6-week whole-body vibration training on the reflex response of the ankle muscles: a randomized controlled trial. Int J Sports Phys Ther.

[21] Cloak R, Nevill AM, Clarke F, Day S, Wyon MA (2010). Vibration training improves balance in unstable ankles. Int J Sports Med.

[22] Nigg B, Federolf PA, von Tscharner V, Nigg S (2012). Unstable shoes: functional concepts and scientific evidence. Footwear Sci.

[23] Nigg B, Hintzen S, Ferber R (2006). Effect of an unstable shoe construction on lower extremity gait characteristics. Clin Biomech.

[24] Osborne MD, Chou L-S, Laskowski ER, Smith J, Kaufman KR (2001). The effect of ankle disk training on muscle reaction time in subjects with a history of ankle sprain. Am J Sports Med.

[25] Boyer KA, Andriacchi TP (2009). Changes in running kinematics and kinetics in response to a rockered shoe intervention. Clin Biomech.

[26] Nigg BM, Emery C, Hiemstra LA (2006). Unstable shoe construction and reduction of pain in osteoarthritis patients. Med Sci Sports Exerc.

[27] Zhang S, Paquette MR, Milner CE, Westlake C, Byrd E, Baumgartner L (2012). An unstable rocker-bottom shoe alters lower extremity biomechanics during level walking. Footwear Sci.

[28] Sierra-Guzmán R, Jiménez J, Ramírez C, Esteban P, Abián-Vicén J (2017). Effects of synchronous whole body vibration training on a soft, unstable surface in athletes with chronic ankle instability. Int J Sports Med.

[29] Hadadi M, Ebrahimi Takamjani I, Ebrahim Mosavi M, Aminian G, Fardipour S, Abbasi F (2017). Cross-cultural adaptation, reliability, and validity of the Persian version of the Cumberland ankle instability tool. Disabil Rehabil.

[30] Mazaheri M, Salavati M, Negahban H, Sohani S, Taghizadeh F, Feizi A (2010). Reliability and validity of the Persian version of foot and ankle ability measure (FAAM) to measure functional limitations in patients with foot and ankle disorders. Osteoarthr Cartil.

[31] Rittweger J (2010). Vibration as an exercise modality: how it may work, and what its potential might be. Eur J Appl Physiol.

[32] Abercromby AF, Amonette WE, Layne CS, McFarlin BK, Hinman MR, Paloski WH (2007). Vibration exposure and biodynamic responses during whole-body vibration training. Med Sci Sports Exerc.

[33] Hertel J, Braham RA, Hale SA, Olmsted-Kramer LC (2006). Simplifying the star excursion balance test: analyses of subjects with and without chronic ankle instability. J Orthop Sports Phys Ther.

[34] Bolgla LA, Keskula DR (1997). Reliability of lower extremity functional performance tests. J Orthop Sports Phys Ther.

[35] Drouin JM, Valovich-mcLeod TC, Shultz SJ, Gansneder BM, Perrin DH (2004). Reliability and validity of the Biodex system 3 pro isokinetic dynamometer velocity, torque and position measurements. Eur J Appl Physiol.

[36] Zouita ABM, Majdoub O, Ferchichi H, Grandy K, Dziri C, Salah FB (2013). The effect of 8-weeks proprioceptive exercise program in postural sway and isokinetic strength of ankle sprains of Tunisian athletes. Ann Phys Rehabil Med.

[37] Hertel J, Miller SJ, Denegar CR (2000). Intratester and intertester reliability during the star excursion balance tests. J Sport Rehabil.

[38] Willems TM, Witvrouw E, Delbaere K, Mahieu N, De Bourdeaudhuij L, De Clercq D (2005). Intrinsic risk factors for inversion ankle sprains in male subjects: a prospective study. Am J Sports Med.

[39] Abraha I, Montedori A (2010). Modified intention to treat reporting in randomised controlled trials: systematic review. BMJ..

[40] Kinzey SJ, Armstrong CW (1998). The reliability of the star-excursion test in assessing dynamic balance. J Orthop Sports Phys Ther.

[41] Sobhani S, Sinaei E, Motealleh A, Hooshyar F, Kashkooli NS, Yoosefinejad AK (2018). Combined effects of whole body vibration and unstable shoes on balance measures in older adults: a randomized clinical trial. Arch Gerontol Geriatr.

[42] Sousa AS, Macedo R, Santos R, Tavares JMR (2013). Influence of wearing an unstable shoe construction on compensatory control of posture. Hum Mov Sci.

[43] Delecluse C, Roelants M, Verschueren S (2003). Strength increase after whole-body vibration compared with resistance training. Med Sci Sports Exerc.

[44] Kaminski T, Buckley B, Powers M, Hubbard T, Ortiz C (2003). Effect of strength and proprioception training on eversion to inversion strength ratios in subjects with unilateral functional ankle instability. Br J Sports Med.

[45] Mohammadi F, Roozdar A (2010). Effects of fatigue due to contraction of evertor muscles on the ankle joint position sense in male soccer players. Am J Sports Med.

